# Effect of abusive supervision on organizational cynicism (Cognitive, affective, behavioral) mediating effect of playing dumb

**DOI:** 10.1371/journal.pone.0284884

**Published:** 2023-04-25

**Authors:** Mahwash Ghafoor Chaudhry, Zartashia Hameed, Fawad Ahmed

**Affiliations:** 1 Department of Management Sciences, University of Wah, Wah, Rawalpindi, Pakistan; 2 Department of Management Sciences, HITEC University, Taxila, Rawalpindi, Pakistan; 3 Examination Department, COMSATS University Islamabad, Wah Campus, Wah, Rawalpindi, Pakistan; University of Castilla-La Mancha: Universidad de Castilla-La Mancha, SPAIN

## Abstract

The purpose of the present study is to examine the link of Abusive Supervision with Organizational Cynicism i.e. Cognitive, Emotional, or Behavioral Cynicism by focusing on the mediating role of Abusive Supervisor’s Knowledge Hiding behavior of Playing Dumb in Higher Education Institutions in Pakistan. Data was collected using questionnaire under the survey research design. The participants included 400 faculty and staff members from Higher Education Institutions in Pakistan. Structural Equation Modelling using SmartPLS is used to test the hypothesized relationships of Abusive Supervision and Knowledge Hiding Behavior of Abusive Supervisors with the faculty and staff’s Organizational Cynicism behaviors. The results indicate that Abusive Supervision is significantly and positively related with faculty and staff’s Cognitive, Emotional, and Behavioral Cynicism. This study also indicates that Knowledge Hiding behavior of Playing Dumb fully mediates the relationship between Abusive Supervision and Cognitive Cynicism and partially mediates the relationship between Abusive Supervision and Behavioral Cynicism. However, Playing Dumb as a knowledge Hiding behaviour does not impact the relationship between Abusive Supervision and Emotional Cynicism. This means that Knowledge Hiding by Playing dumb contributes to the adverse effect of Abusive Supervision, leading to increased Cognitive and Behavioral Cynicism. This study contributes to literature on Organizational Cynicism and Abusive Supervision by studying the link between them and effect of Abusive Supervisor’s Knowledge Hiding behavior of Playing Dumb as a mediator. The study also indicates that Abusive Supervision characterized by Knowledge Hiding behavior of Playing Dumb is really a problem in Higher Education Institutions in Pakistan. This study holds importance for top management in Higher Education Institutions in to curb the negative effects of Abusive Supervision by developing a policy framework that ensures prevention of Organizational Cynicism in faculty and staff. Moreover, the policy guidelines should ensure that control of essential resources such as Knowledge in the hands of the Abusive leaders should not be misused, causing Organizational Cynicism and eventually leading to problems such as turnover and psychological and behavioral issues among faculty and staff in Higher Education Institutions of Pakistan.

## Introduction

The concept of Leadership evolved and developed with changes in the organization and environment [1] over the past centuries. Organizations need leaders with the ability and skill to help organizations in their existence, growth, development, and goal achievement [2]. However, an expanding amount of literature focuses on unethical Leadership, which has led to studies on abusive supervision [3, 4] suggesting that Leadership is not always a force for good [5] Also, organizations are recently facing Abusive Supervision (AS) [6] which affects workers’ commitment levels or extra-role activities [7]. According to Tepper [8], AS results in job and life dissatisfaction, reduced normative and affective commitment, increased psychological distress, and even leads to job turnover in subordinates. Abusive supervisor practices tyranny through verbal and non-verbal Behavior [8], due to which employees lose their passion and commitment to their job [6] and negatively lowers employee motivation. Organization Cynicism (OC) is an essential outcome of Leadership [9, 10]. It is a person’s unfavourable attitude about their organization, which includes beliefs that contribute to a lack of organizational integrity, unfavourable feelings, and a propensity for critical and derogatory behaviour [11].

People in Pakistan are accustomed to a dictatorial and power-driven leadership style [12], as Abusive Leadership is on the rise in Pakistan [13]. Workplace mistreatment is a prevalent and unreported dilemma in Pakistan [14] in higher education institutions, particularly witnessing workplace issues such as maltreatment, expulsion, uncivil behaviour etc. [15]. The rationale behind the focus of the present study on the AS experienced by the teaching faculty in higher education in Pakistan is to reveal the impact the leaders’ abuse has on the teaching faculty’s cynicism behaviour. Teachers, the primary stakeholders, play a key role in educational institutions [16]. However, their motivation and enthusiasm are lost due to a lack of support from superiors and they become dissatisfied with their profession [16]. Apart from insulting and criticizing, AS also abuses employees by holding the needed information [8, 17]. Therefore, AS is negatively related to information exchange [18] and positively related to Knowledge Hiding (KH) [19], which is defined in literature as deliberate hiding of knowledge from the one who asks for it [20].

### Contribution and originality

The present explores the effect of AS on OC and the mediating effect of KH behaviour of Playing Dumb (PD) in Higher Education Institutions (HEIs) in Pakistan and hence has made several contributions to the body of knowledge on Abusive Leadership and its consequences on the employee mental well being and performance. First, this study discusses the role of abusive behaviour from the perspective of employees’ OC. Second, this study describes KH Behavior of PD as a mechanism that channels the influence of Abusive Leadership on the Cynicism Behaviors of employees in the Education Sector of Pakistan. Third, it contributes to the literature by addressing various research gaps. First, there is a dearth of research on how abusive supervisory practices result in knowledge hoarding [21]. Even though academics and practitioners have started to show interest in new knowledge management principles, such as Knowledge Hiding (KH), there is still a need for adequate empirical study. The phenomenon of KH is usually taken as a unitary construct and can be best understood as a construct consisting of three facets.

Moreover, little is known about the targets, perpetrators, outcomes, and context of KH [22] and thus can be considered a concept still in its infancy [23] The prospect of the Leaders-signal KH triggers, framework, and conditions that lead to various signal perceptions and consequences across cultures is a promising area for future research [24] Limited research studies exist on the link between information hoarding and AS [25]. However, most research focuses on the connection between knowledge-sharing behaviour and supervisor abuse, which differs from knowledge-hoarding behaviour regarding the employee’s goal and aim [26]. Past studies focused on the impact of AS on KH by the employees [27]. Research suggests that more diverse leadership styles need to be studied concerning KH and its antecedents [28].

The novelty of the present study lies in exploring the impact of AS on the cognitive, emotional and behavioral dimensions of OC and the mediating role of knowledge hiding behaviour of "Playing Dumb", as evident from the research gap discussed earlier. Most of the studies in the past explored the knowledge-hiding behaviour of employees, but the novel aspect of the present research is investigating the effect of the knowledge-hiding behaviour of abusive Leadership on employee organizational cynicism. Faculty retention has become a significant challenge for institutions in Pakistan [29] with a dramatic rise in switching trend among educational institutions in the last decade [30, 31] as abusive supervision impacts psychological well being and turnover intentions of employees [32].

This study aims to solve the problem of employee turnover due to AS experienced by the faculty in the HEIs in Pakistan by focusing on the role of AS in OC. The present study investigates the influence of AS on OC (Cognitive, Affective, Behavioral) and the mediating function of KH behaviour of PD.

The literature review section presents a review of pertinent research and supporting theories. The research methodology follows next, then the analysis, and lastly, the explanation of the results. The final section discusses management implications, constraints, and recommendations for future research.

## Review of literature

### Abusive supervision

Abusive supervisions portray antagonistic activities toward subordinates and mal-treat, criticize, intimate, aggressively stare, ridicule, and require information from them [8, 17]. Kelly et al. [33] attested that the past decade had recorded a blast of interest and inquiry about Abusive Supervision. Such behaviors regularly incorporate scorning and mortifying subordinates in the open, refusing to talk with subordinates, or corrupting subordinates. AS leads to counterproductive work conduct [8] and has been explored as an antecedent to a few negative working environments results [34, 35].

### Organizational cynicism

Organizational Cynicism (OC) refers to the absence of employees’ sense of justice, assurance, fairness, and honesty toward the company where they work [36]. OC is not a personality trait but a learned reaction [37]. OC is a general and particular attitude characterized by anger, despondency, disillusionment, and a tendency to distrust individuals, groups, ideologies, social abilities, or institutions [38]. OC is a three-dimensional negative attitude toward one’s organization. The first dimension is Cognitive Cynicism (CC) which is the perception or belief that an organization lacks honesty and integrity and disbelief in the sincerity of people’s and the organization’s motives. Second is Emotional Cynicism (EC) which, apart from opinions and beliefs, includes strong emotional responses towards the organization, i.e. condemnation, hatred, anger, disgust, embarrassment etc. The third and last dimension of OC is Behavioral Cynicism (BC), which represents negative behaviours about an organization, i.e. criticism, satirical humour, negative assertions etc. [39, 40]. OC is a complicated phenomenon that leads to the conviction that the organization is unfair.

#### Cognitive cynicism (CC)

Belief in the organization’s lack of integrity is the first cognitive dimension. The notion that an organization’s actions lack fairness, honesty, and sincerity constitutes OC’s cognitive (belief) component. Employees’ scepticism of their companies is called the cognitive dimension [41]. They believe their organizational procedures betray them due to these beliefs [11].

#### Emotional cynicism (EC)

The second dimension of OC is emotional/sentimental reactions to the organization. Strong emotional responses against the organization make up OC’s sensitive/emotional part. The emotional dimension comprises emotional responses, including fear, embarrassment, wrath, disappointment, or rage/pessimism [42]. OC has a strong emotional component, including disdain, resentment, boredom, and embarrassment [36].

#### Behavioral cynicism (BC)

The final dimension concerns unfavourable inclinations, particularly demeaning attitudes. The final aspect of OC, the behavioral dimension, comprises unfavourable and frequently critical attitudes. Strong criticism of the organization is the most noticeable behavioral tendency. The behavioral dimension includes the organization’s harsh employee comments, such as condescension, denigration, and belittling [43].

The present study can be viewed in light of theories and models presented by past researchers. Social Exchange Theory explains the link between KH and AS. According to this theory, Individuals may change their attitudes and behaviors depending on how they are treated or the need for reciprocity [44]. According to the Reactance Theory proposed by Brehm [45], an individual tends to preserve their sense of empowerment and autonomy when threatened or feel a loss of control by reacting against their supervisor s by reducing work efforts at work. The unpleasant motivational state of this reactance comes forth in the form of cognitive and behavioral efforts by the employees to sustain their autonomy along with experiences of emotion such as feeling uncomfortable, aggressive, anger [46, 47]. The Affective Event Theory claims that AS stifles employee creativity because work events, especially negative ones, impact employees’ emotions and cause various reactions regarding feelings and behaviors [48]. As an extension of AET to AS literature, a study suggests that AS links the event-emotion-behaviour process [49]. The Theory of Followership explains the effect of the leadership process on followers involving the role of subordinates and subordinates’ behaviour directed by the supervisors. Associated outcomes [50] and consider abusive behaviour as self-serving and unjust, leading to feelings and reactions to the negative Behaviour [51]. According to the EVLN model, exit, voice, loyalty, and neglect (EVLN) help combine behavioral intents as responses to AS. It covers potential behavioral reactions when faced with unsatisfactory work conditions [52].

Literature finds AS to be a leading and significant cause of various organizational outcomes such as emotional exhaustion [53], negative effects on worker job attitudes such as reduced job satisfaction [8, 54], lower job involvement, negative wellbeing and perceived organizational support [54], workplace deviance [55], reduced task performance [56], lower normative and affective commitment, work-life conflict [8] and psychological distress [8, 57] lesser engagement in Organizational Citizenship Behavior [7, 56]. The past literature indicates that abusive supervision is negatively associated with a negative organization and employee performance outcomes [58]. AS makes workers feel inferior and degraded, which eventually results in Organizational Cynicism [59], yet none of the research to researchers’ knowledge has explored the impact of AS on the dimensions of the OC. The present study will help to assess the issues of causality as suggested by previous research on abusive supervision [58] on Organizational Cynicism dimensions. The present study explores the AS role in affecting all the dimensions of OC, namely CC, EC, and BC experienced by faculty in HEIs in Pakistan in the hypothesis given below

H1: Abusive Supervision has a significant impact on Cognitive Cynicism.
H2: Abusive Supervision has a significant impact on Emotional Cynicism.
H3: Abusive Supervision has a significant impact on Behavioral Cynicism.

#### Playing dumb (PD)

Even though extant writing has comprehensive studies examining why, how, and when individuals share their information, it is almost silent on why, how, and when individuals cover up their information. One such work factor that hinders organizational objectives is the subordinate’s Knowledge Hiding (KH) conduct, and AS is considered a barrier to Knowledge sharing as it has characteristics of discretionary behaviors [18].

KH is one of the unfavourable behavioral effects; however, most workers under AS prefer to neglect this conduct. Many practitioners and academics, however, believe that sharing information and keeping it to oneself are two ends of the same continuum. KH comprises three distinct behaviors: playing dumb, evasive Hiding, and rationalized Hiding [60]. When hiding rationally, the offender explains not divulging the desired information (e.g., because it is confidential). When playing dumb, the hider pretends to be unaware of the desired information. Deception is included in evasive Hiding and playing dumb, but not rationalized hiding [24, 61]. Connelly & Zweig [62] discovered that rationalized hiding was unrelated to retribution expectations and intentions, whereas Evasive Hiding and PD were. Similar findings were made by Zhao et al. [61] who discovered that ostracism predicted Evasive Hiding and PD but not rationalized Hiding.

According to Conservation of Resource (COR) Theory [63], Dr Steven E. Hobfoll explains that AS behaviour affects the resources such as required knowledge needed by the subordinates. Abusive supervisors conserve their resources [64] and reduce the employees’ control and ability to affect their actions, changes, and situations by reducing their access and control over the resources (i.e., Knowledge) required to perform their jobs [65].

Numerous studies on AS consider it a root cause of several unfavourable workplace outcomes [58, 66] Past literature found various variables that moderated some of the deleterious effects of abusive supervision, such as Job Mobility [8], Research Design, National Culture and Demographic Characteristics [67], Power distance [68], meaning at work [69], social support [35, 70] yet limited research exists to assess the role of KH, the purposeful decision to suppress information and knowledge needed by others [60], as moderator on relationship between AS and Individual and organizational knowledge outcomes.

Most past studies focused mainly on sharing knowledge rather than KH behaviors [71]. Few studies have explored the relationship between KH and Cynicism, such as KH negatively affects employees’ thriving through psychological safety, reliant on the influence of OC [72] and moderating effect of employee cynicism on the relationship between the knowledge Hiding and tolerance to workplace incivility [73]. Past research studies have not explored the role of knowledge-hiding behaviours as moderators on the relationship between AS and dimensions of OC. Therefore, in the present study, we concentrated on the deceptive KH behaviors of PD as induced by interpersonal conflict with co-workers, in keeping with Connelly [22] view that specific knowledge concealing features may be explored independently. The study aims to investigate the impact of PD and knowledge concealment behaviour on the association between AS and Cognitive, Emotional, and Behavioral Cynicism in light of the above mentioned arguments from the literature.

H4: Playing Dumb mediates the relationship between Abusive Supervision and Cognitive Cynicism
H5: Playing Dumb mediates the relationship between Abusive Supervision and Emotional Cynicism
H6: Playing Dumb mediates the relationship between Abusive Supervision and Behavioral Cynicism

## Materials and methods

### Procedure and participants

The present research uses a quantitative approach using a survey method to explore the effect of AS on three dimensions of OC: Cognitive, Emotional, and Behavioral Cynicism, using information gathered from employees of HEIs in Pakistan. The cover letter ensures and informs respondents about the study’s nature, confidentiality, and goal. The study includes educated adults working as faculty and staff in HEIs in Pakistan. The cover letter attached to the questionnaire ensures respondents’ confidentiality and willingness to participate in the survey. The statement of the cover letter stated that the respondents were requested to participate in the survey if they were willing to be a part of it else; they had the right not to participate in the survey in the details mentioned in the cover letter section of the survey questionnaire. The Ethical/ Biosciences Research Committee of the University of Wah (ERC), Pakistan, approved this study and since cover letter attached ensures willingness of the respondents to participate in the study. Therefore the ERC states that there is no need for any written or verbal formal consent from the respondents in this study.

The questionnaire prepared on google Forms was distributed through email and other social media applications such as Instagram and WhatsApp. A total of 400 faculty and staff members participated in the Questionnaire Survey, and 35 questionnaires were discarded due to missing data leaving a total of 365 questionnaires included in the final analysis. Among the 340 respondents, 55.6.% were male, and 44.4% were female. In terms of age, 20 (5.9%) are less than 25 years, 153 (45%) are between 25 to 35 years of age, 147(43.2%) are between 36 to 45 years of age, and 20 (5.9%) respondents are between 46 to 55 years of age. In terms of designation, 137 (40.3%) respondents are lecturers, 33 (9.7%) are Assistant Professors, 32 (9.4%) are associate professors, and 138 (40.6%) worked on other designations. The study participant with less than two years of experience are 110 (32.4), with experience between 2 to 5 years 42 (12.4%), with experience between 6 to 10 years 68 (20%), and those with experience of more than 10 years are 120 (35.3%).

### Measures and instruments

A survey questionnaire with scale items measured on a five-point Likert scale (Strongly Disagree represented by 5 and Strongly Agree represented by 1 was used to gather information from the respondents, including the following measures.

#### Independent variable

*Abusive Supervision (AS)*. AS is measured using 15 items adopted from Tepper [8]. The instruments used to develop the scale for AS by Tepper [8] captured the nonphysical abuse in other relationships. The 15 items used to measure AS are such as my boss ridicules me. He tells me my thoughts or feelings are stupid. etc. The respondents rated their level of agreement with statements on a 5-point Likert scale with optional ranging from Strongly Disagree (1) to Strongly Agree (5). The Cronbach’s alpha for the scale of AS is 0.949.

#### Dependent variable

*Organizational Cynicism (OC)*. A 13-item OC Survey developed by Brande [42] measures OC comprising of three dimensions: Cognitive, Emotional, and Behavioral Cynicism. CC, defined as the belief that an organization lacks justice and integrity, is measured by four items. The second dimension of EC reflects powerful negative emotional reactions in anger, distress revulsions, etc., measured using four statements of emotional dimensional of the OC Scale. BC, the third dimension of OC reflecting critical and disparaging behaviour in the form of criticism, unfavourable gestural behaviors, and sarcasm, is measured using five statements of behavioral dimension. The items are measured on a scale of 1 (strongly disagree) to 5 (strongly agree), and questions 2, 3, and 4 of the Emotional Cynicism are measured on a scale of never “1” to often “5”. The Cronbach’s alpha value of the scale of CC is 0.895, EC is 0.876, and BC is 0.917

#### Mediating variable

*Playing Dumb (PD)*. PD is an essential dimension of KH behaviour known as PD. It involves deception describing behaviour in which the hider pretends that he/she is ignorant about relevant information. This dimension is assessed using four items on a 5-point Likert scale from 1 (Strongly Disagree) to 5 (Strongly Agree). The Cronbach’s alpha value for the scale of PD is 0.949.

### Data analysis

SPSS 22 and Smart PLS 4.0 are used to analyze data. Smart PLS 4.0 is used for confirmatory factor analysis, path analysis, and structural equation modelling. Confirmatory Factor Analysis (CFA) is performed using SMART PLS 4.0, and Confirmatory Factor Analysis is conducted.

### Assessment of the measurement model

The results in Table 1 show that Cronbach’s Alpha and Composite Reliability, used to establish the model’s reliability, are > 0.8 and 0.9, respectively [74, 75]. Collinearity Statistical results of VIF show that the recommended common threshold of VIF value is 10 [79] and VIF value is 5 [76]. No pathological collinearity and common method bias are found in the results [77].

**Table 1:** Reliability, composite reliability, and validity analysis.

Variable and Constructs	Loadings	Alpha	CR	rho_A	AVE	VIF
**Abusive Supervision (AS)**		0.950	0.955	0.953	0.590	
**AS1**	0.722					2.273
**AS2**	0.785					2.602
**AS3**	0.692					2.536
**AS4**	0.768					2.478
**AS5**	0.774					2.859
**AS6**	0.773					2.836
**AS7**	0.621					1.807
**AS8**	0.779					3.643
**AS9**	0.777					3.254
**AS10**	0.785					3.476
**AS11**	0.755					3.968
**AS12**	0.843					4.141
**AS13**	0.724					2.514
**AS14**	0.849					3.965
**AS15**	0.838					3.849
**Behavioral Cynicism (BC)**		0.862	0.901	0.870	0.646	
**BC1**	0.820					1.888
**BC2**	0.738					1.721
**BC3**	0.800					2.038
**BC4**	0.892					3.000
**BC5**	0.759					1.894
**Cognitive Cynicism (CC)**		0.838	0.851	0.848	0.673	
**CC1**	0.820					2.444
**CC2**	0.738					1.987
**CC3**	0.800					1.652
**CC4**	0.892					1.754
**Emotional Cynicism (EC)**		0.801	0.870	0.811	0.625	
**EC1**	0.820					1.556
**EC2**	0.738					1.682
**EC3**	0.800					1.562
**EC4**	0.892					1.655
**Playing Dumb (PD)**		0.838	0.892	0.845	0.674	
**PD1**	0.865					2.385
**PD2**	0.804					1.924
**PD3**	0.834					1.781
**PD4**	0.778					1.754

Table 1 above shows that Cronbach’s values are > 0.7, and composite reliability is > 0.8. Using three criteria to establish Convergent Validity, each factor loading greater than 0.5 [78], composite reliability greater than 0.70, and Average Variance Extracted (AVE) value should be more than 0.50 [79, 80] as in results in Table 1. According to Aiken [81] the value of VIF must be less than 10 to 5 thresholds. So, it can be seen in Table 1 that there is no issue of Multicollinearity as the values of VIF are within the threshold limits.

According to Fornell and Larcker [80] the root of one construct should be greater than its correlation with all the others. Fornell -Larcker Criterion [8]) is considered one of the most popular techniques to check Discriminant Validity, measuring the extent to which the constructs are distinct. It can be seen in Table 2 that the Square of AVE of all constructs is higher than the values underneath them, which are the correlation of each particular construct with other constructs. It can be seen that, in all. According to [79] the square root of the average variance extracted ought to be higher than the values of the correlations. The results given below in the table meet the criteria of both Fornell and Larcker [80] and Henseler et al. [79] to establish Discriminant Validity.

**Table 2:** Discriminant validity.

	Abusive Supervision (AS)	Behavioral Cynicism (BC)	Cognitive Cynicism (CC)	Emotional Cynicism (EC)	Playing Dumb (PD)
**Abusive Supervision (AS)**	0.768				
**Behavioral Cynicism (BC)**	0.566	0.803			
**Cognitive Cynicism (CC)**	0.383	0.460	0.821		
**Emotional Cynicism (EC)**	0.640	0.525	0.404	0.791	
**Playing Dumb (PD)**	0.676	0.534	0.563	0.496	0.821

HTMT is a method for estimating the correlation between two latent variables. It measures the extent of similarity between the latent variables, established for values less than 0.9 [78, 79] or less than 0.85 [74] as shown in Table 3.

**Table 3:** HTMT test findings.

	AS	BC	CC	EC	PD
**AS**					
**BC**	0.615				
**CC**	0.421	0.534			
**EC**	0.712	0.626	0.481		
**PD**	0.748	0.618	0.665	0.594	0.000

#### Assessment of structural model

R^2^ also referred to as in sample predictive power, represents the variance explained by each indigenous variable in the model and measures the model’s explanatory power. The model fitness was assessed using R^2^ (78) R^2^ values can be substantial, moderate, and weak depending on values of 0.75, 0.50, and 0.25, respectively. The table below shows that the combined effect of KH behaviour of PD with AS explains 36% variation in CC, 32% variation in EC 42% variation in BC, whereas AS explains 45% variation in KH behaviour of PD. Q square values are all greater than zero, as shown in Table 4.

**Table 4:** Structural model assessment.

	Q square	R Square
**Cognitive Cynicism (CC)**	0.363	0.363
**Emotional Cynicism (EC)**	0.317	0.317
**Behavioral Cynicism (BC)**	0.418	0.418
**Playing Dumb (PD)**	0.457	0.457

#### Hypothesis testing (Direct effect)

The present study made three direct hypotheses: H1, H2, and H3. The H1 Hypothesis test the proposed relationship between AS and CC The results of the first hypothesis indicate that AS positively impacts the CC experienced by the Teachers and Staff in the HEIs in Pakistan. The result values of B = 0.522, t = 8.313, and P value = 0.000 (less than P <0.001) reveal that AS positively impacts CC. Hence the H1 hypothesis is accepted.

**Fig 1:**
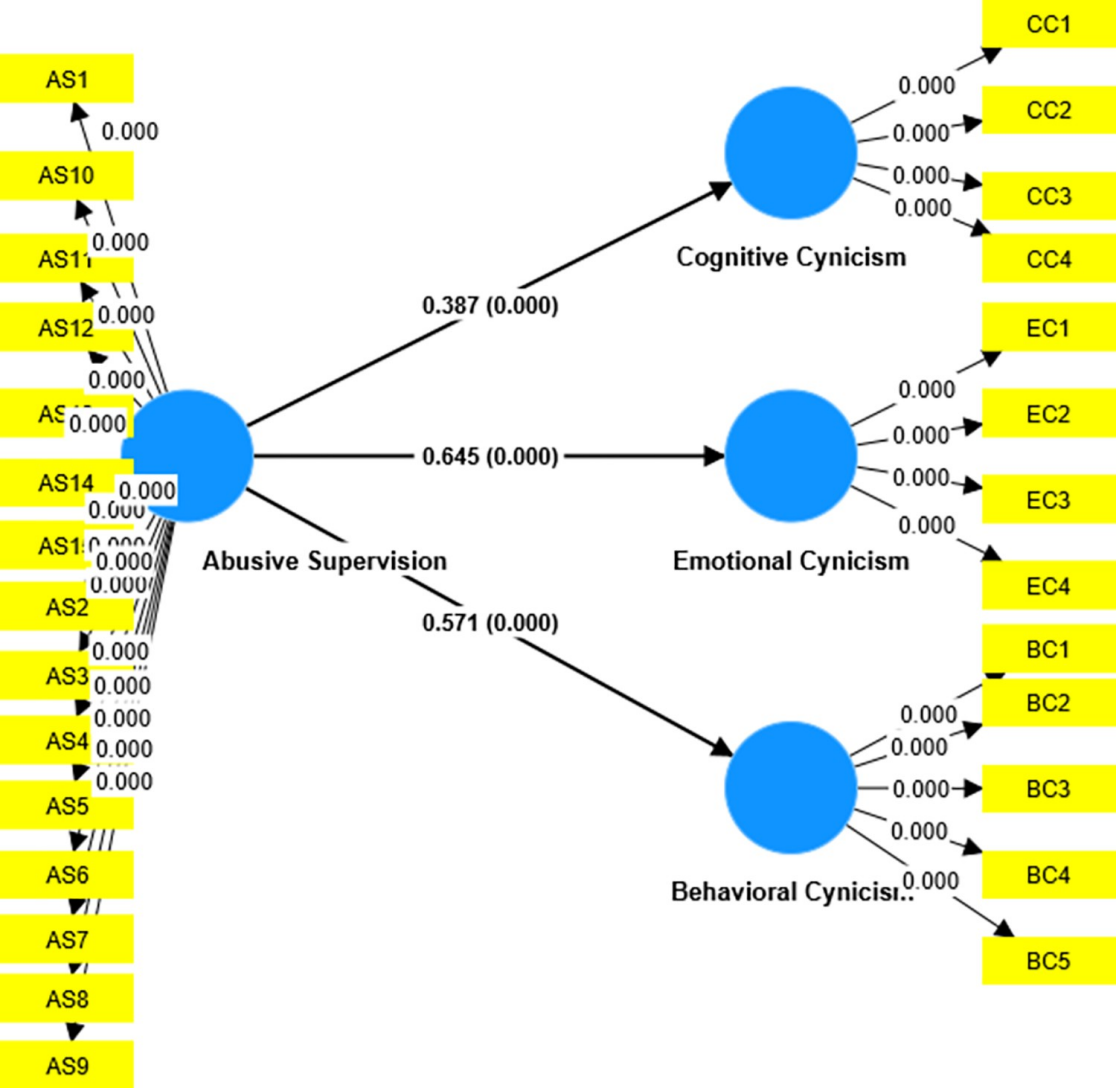
SEM model.

H2 evaluates if AS positively and significantly impacts EC. The results of B = 0.325, t = 4.958, and P value = 0.000 (less than P<0.001) indicate that the findings support the H2 hypothesis. The H3 hypothesis tests the impact of AS on BC. The Bootstrapping results indicate that given the result values of B = 0.657, t = 13.579, and P value = 0.000 (less than P = 0.001), it supports the H3 and establishes the finding that AS does positively and significantly affects BC. The path coefficient results are given in Table 5, and Fig 1 shows the SEM results of the basic model and the impact of AS on CC, EC and BC.

**Table 5:** Path coefficients (Direct effects).

Hypothesis	Relationship	B	T	RSquare	P	Decision
**H1**	AS → CC	0.387	7.209	0.150	0.000	Yes
**H2**	AS→ EC	0.645	14.497	0.416	0.000	Yes
**H3**	AS→ BC	0.571	11.545	0.325	0.000	Yes

#### Playing Dumb (PD) mediating effect

**Fig 2:**
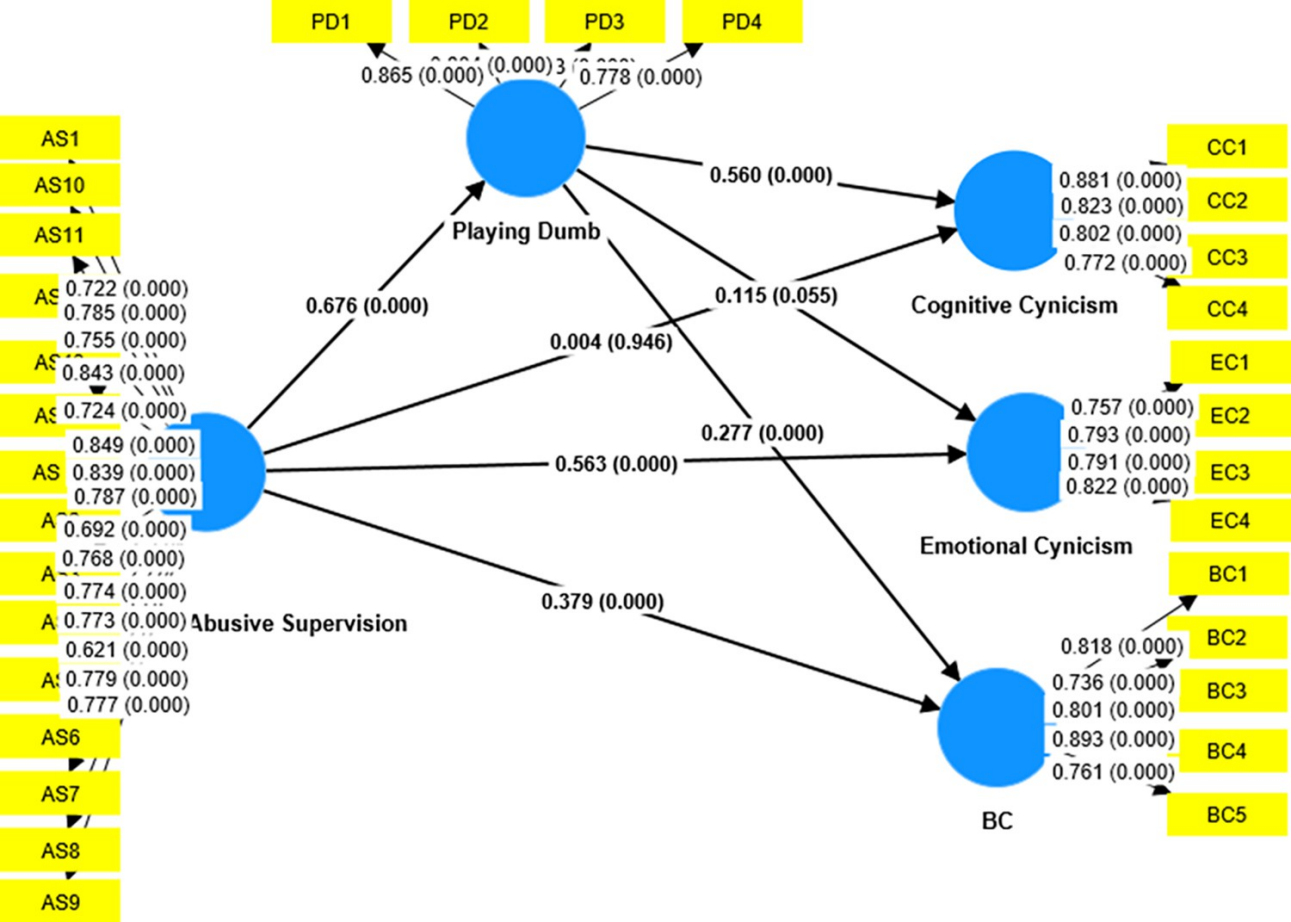
Mediation model.

The current study aims to determine if knowledge concealment practices mediate the associations between AS and the three dimensions of OC, namely Cognitive, Emotional, and Behavioral Cynicism. The mediation results illustrate that KH mediates the association between AS and CC. The results of the indirect effects in the Table 6 given below shows that there exists a strong collateral impact of AS on CC through KH Behavior (B = 0.326, t = 6.880 and p-value = 0.00), and a direct effect of AS on CC becomes insignificant in the presence of the KH (B = 0.056, t = 0.884 and p-value = 0.377. Hence H4a is accepted as KH fully mediates the connection among AS on Cognitive Supervision. An insignificant indirect effect of AS on EC through KH (B = 0.018, t = 0.405, and p value = 0.686) proves that KH does not mediate the connection between AS and EC. So, Hypothesis H4b is rejected. A significant indirect effect of AS exists on BC though KH (B = 0.205, t = 4.327, and p-value = 0.000). The direct effect of AS on BC is also significant in the presence of KH (B = 0.343, t = 4.781, and p value = 0.000), which means that KH partially mediates the relationship between AS and BC. The results of the mediation effect of KH on the relationship between AS and OC Dimensions are given below in Table 6. Fig 2, given below, shows the results of the mediation results of the model.

**Table 6:** Mediation result analysis with playing dumb as a mediator.

Total Effect			Direct Effect				Indirect Effect				Percentile bootstrap 95%confidence Interval	
Coefficients	T Value	p-value	Coefficient	T- value	p-value	Hypothesis	Coefficients	SE	T-value	p-value	Lower	Upper
0.383	6.904	0.000	0.004	0.067	0.946	H3a: AS→KH→CC	0.379	0.045	8.444	0.000	0.294	0.470
0.641	14.233	0.000	0.563	9.601	0.000	H3b: AS→KH→EC	0.078	0.042	1.853	0.064	-0.002	0.166
0.566	11.260	0.000	0.379	5.902	0.000	H3c:AS→KH→BC	0.187	0.044	4.242	0.000	0.101	0.274

## Discussion

The present study is one of the empirical investigations attempting to explore the construct of PD, a dimension of KH Behavior as a mediator. The study’s findings suggest that AS leads to different forms of cynicism in faculty and staff in HEIs of Pakistan. Employees perceive ill-treatment or other forms of abuse from their supervisors, which leads to cynical behaviour. AS is considered an incident of many negative outcomes in the organization [35] such as KH [82] and OC [83]. The study finds that AS experienced by the faculty and staff in HEIs of Pakistan positively and significantly affects Cognitive, Emotional, and Behavioral Cynicism based on the findings of hypothesis H1, H2, and H3. The studies on other types of leadership styles support the current study’s findings. Such as a study has found similar results in a military educational setting, demonstrating the positive relationship between toxic relationships and OC [84]. Ethical leadership behaviour of faculty administrators in universities is considered a significant predictor of OC and has a negative impact on academics’ Organizational Cynicism Behaviors [85]. Narcissistic Leadership positively affects OC [86, 87], specifically BC [88] and CC [89].

The study also finds that KH behaviors of PD fully mediate the relationship between AS on CC and Partially mediate the relationship between AS on Behavioral Cynicism. However, the association between AS and EC is not mediated by the KH behavior of PD. The finding is supported by evidence from past studies, in which the employees feel psychologically insecure and are less likely to thrive at work in the presence of KH behaviors [72]. KH behaviors are triggered by the deviant and detached behaviour of leaders and the motivations of employees [90].

Retaliation behaviour supports the findings explained under the Leader Membership Exchange Theory, which explains that the relationship between leader and subordinate depends on the nature of the social exchange between them [91]. According to this theory, an individual performs harmful acts in response to injustice and ill-treatment. The negative emotion in response to the negative event is due to the individual as causal attribution for the precipitating event. According to Connelly and Zweig [60] PD relates to retaliation expectations and intentions among individuals. A leader’s KH behaviour reduces employee creativity and job performance through feedback avoidance behaviour [92]. Un-pledged hiding behaviors increase the impact of abusive leadership on Cognitive and Emotional Cynicism rather than BC. The findings indicate that AS leads to a transnational cynicism behaviour composed of beliefs, affect, and behavioral tendencies, thus supporting the mediation role of KH behaviour of PD on abusive leadership and OC behaviors. Drawing on the Social Exchange Theory [44], Psychological Ownership Theory [93] and Norms of Reciprocity [94].

According to a study, AS is more likely to depict KH behaviour [19], and employees working under the Leaders signaling KH, such as PD, a behavioral dimension of hiding knowledge, are less satisfied with jobs, feel less empowered and more to experience turnover intentions [24]. The KH behaviour mediates this impact by contributing toward holding cynical beliefs and experiencing cynical behaviour against the organization.KH is identified as a crucial factor by which AS reduces employees’ capacity for creativity and intensifies workers unfavourable views of reciprocity, making them more susceptible to negative social interaction [95]. Another study reports the results that leaders’ knowledge hiding conduct makes employees’ behavior worse, which hinders their ability to be creative and perform well at work [92].

## Conclusion

The findings of the present research indicate that AS led by the heads of departments in HEIs in Pakistan does contribute significantly towards the OC experienced by the faculty and staff working under them. They experience Emotional, Cognitive and Behavioral Cynicism when they work under the supervision of an abusive supervisor. Supervisors are usually considered proxies for the organizations; therefore, their abusive actions may result in a wrong opinion of the company among employees. The present study establishes that AS contributes to strong negative feelings and emotions towards supervisors and jobs. Carefully implementing a transactional leadership style may help with employee retention difficulties faced by the HEI in Pakistan [96]. The KH behaviors of PD (pretending not to know) are considered intentional and deceptive [22]. An abusive leader, by playing dumb act as if he/she does not know anything, positively contributes towards the organization’s cynicism experienced by the employee by making the behaviour of faculty and staff working under the abusive department head more unpredictable and erratic. This is so because by playing dumb and not sharing the required information, the abusive supervisor affects the CC in the employee, and the employee develops the belief that the institution lacks integrity, justice and honesty. The playing dumb knowledge hiding behaviour by an AS partially affects BC in Faculty, and Staff expressed in the form of sarcastic humour, criticism of the organization, unpleasant non-verbal behaviour, negative interpretations etc. The partial mediation of PD on the relationship between AS and BC means there is not only a significant relationship between PD of Abusive supervisor and Behavioral Cynicism, but there are some other attributes of AS other than KH behaviour of PD that significantly affect the Behavioral Cynicism in employees. However, the KH behaviour of an abusive supervisor by PD does not affect employees’ EC. Knowledge hiding by the abusive supervisor by pretending or acting not to know anything does not trigger the emotional or sentimental emotions of anger, anxiety or tension in employees.

### Theoretical implications

By embedding OC in the nomological network of less empirically investigated and explored variables, the present study highlights the importance of AS for psychological strain and behavioral responses enhanced under the deceptive KH behaviour of "Playing Dumb" (PD), where the hider pretends not to have the relevant information requested by the seeker. AS can lead to various negative outcomes that can impact the growth and profitability of organizations, especially knowledge-intensive organizations, mainly due to the interpersonal animosities and cynicism among employees involving intentional KH [71]. Abusive leadership trickles down though out the organization and eventually affects the employees’ perceptions, emotions, and behaviors [52, 97]. The present study significantly contributes to the literature on AS and OC by exploring the impact of AS on all of the dimensions of OC i.e. Behavioral, Cognitive and Emotional Cynicism. Therefore, organizations, where employees experience cynicism due to AS and KH are less likely to give the desired performance due to employee turnover. The present study only focuses on the KH behaviour of PD, to study single hiding behaviour individually [20]. The present study contributes to the existing literature on AS by studying the extent to which knowledge-hiding behaviour of AS in HEIs triggers Cognitive, Behavioral and Emotional Cynicism experienced by faculty and staff in these institutions. Most past studies have explored the KH behaviour of employees as a result of AS behaviour, but the present study, unlike the past research works, studies KH behaviour of AS by playing dumb on the Cognitive, Emotional and Behavioral aspects of the OC experienced by them. According to literature the KH behaviors relate differently to various responses to employee’s interpersonal and relationship conflicts [98, 99].

### Practical implications

The present study holds several practical implications for management and employees in HEIs to effectively overcome cynicism which eventually leads towards employee turnover, as explained by Equity Theory and Social Exchange Theory. These theories explain the association between organizational cynicism and organisational turnover intention [100]. The abusive behaviour of the supervisor hold consequences not only for the employees but for the organizations, as high turnover affects the reputation and performance of the HEIs. Regular training should be conducted in HEI to guide the heads/supervisors to curtail abusive supervisory behaviour and play their role as knowledge facilitators, not hiders. HEI institutions need to set policies to deliver the required knowledge and information to employees from multiple channels to avoid OC due to knowledge hiding behaviour of supervisors/heads in HEIs in Pakistan. A centralized knowledge management system allowing free knowledge sharing and creation will enable faculty and staff to manage day-to-day business activities. This free flow of information will not only improve faculty and staff access to relevant information but also ensure task achievement by improving organizational efficiency and effectiveness as a whole. It will also reduce the delays in getting approval from various hierarchical levels, a common feature of the bureaucratic organizational structure in HEI in Pakistan.

### Societal implications

The study holds societal implications by suggesting that the abusive behaviour of an individual can cause mistrust and contempt among other people and society by simply hiding the information that he/she needs to perform well. Employee cynicism spreads like a viral pandemic in society when the cynics share what they experience, i.e. psychological distress, frustration, anxiety, tension, lack of trust, integrity and feeling of justice etc. while working under an Abusive leader. The bad experiences of one can contaminate the whole society when they share their cynical opinion with people around them or return what they get. This creates a negative vibe in society which eventually affects all the aspects of the individual personality, including creativity, motivation, dedication, and loyalty; in short, all the positive aspects of society lead to a more common bad than a common good the society. Thus, AS must be dealt not only for the organizational performance but also for a healthy and progressive society. Psychological counselling of employees and supervisors at individual and organizational levels will help them overcome this negativity and prevent it from spreading by understanding the dire consequences this Cynicism can have for society.

### Limitations

Even though the current study adds to the body of knowledge on the cynical reactions of employees to AS through the mediating effect of playing dumb, it also has certain limitations. The data is collected from a small sample in size and cross-sectional nature constitute the first limitation. A better and more reliable finding can be made with more data, gathered and compared across public and private sector schools, colleges and educational institutions other than HEIs in Pakistan. Various dimensions of OC are examined in this study to determine how AS affects them. It is possible to compare how various leadership styles affect the employees’ level of OC, which may lead to a better understanding of promoting the kind of leadership styles which eliminate or minimize the adverse effects on employees’ OC. Also, the present study focuses on HEIs only. The generalization of the finding can be established by conducting studies in other sectors also. The present study has focused on the impact of the KH behaviour of PD on various dimensions of OC. Other behaviors and constructs as mediators and moderators could be explored in future studies on the role of Abusive supervisor in various industrial and societal contexts. Another limitation of the study is the using only one KH Behavior as a mediator. Future studies can explore this relationship under other KH behaviors and individual and organizational factors.
